# A Fully Online Research Practicum Curriculum for Undergraduate Medical Students: A Protocol Paper

**DOI:** 10.7759/cureus.31901

**Published:** 2022-11-26

**Authors:** Robin J Jacobs, Joshua Costin

**Affiliations:** 1 Department of Medical Education, Research, Health Informatics, Nova Southeastern University Dr. Kiran C. Patel College of Osteopathic Medicine, Fort Lauderdale, USA; 2 Department of Medical Education, Nova Southeastern University Dr. Kiran C. Patel College of Allopathic Medicine, Fort Lauderdale, USA

**Keywords:** research training, teaching online, education technology, distance learning, medical student, curriculum, undergraduate medical education, preclinical, research practicum

## Abstract

Introduction: Research is a critically important skill for medical trainees that helps physicians to advance the state of science and provide optimal healthcare to patients. The number of physician-scientists is decreasing. Competing priorities, limited research faculty, shrinking research budgets, and meeting accreditation standards limit the amount of time and effort needed to train undergraduate medical students sufficiently for them to engage in independent research activities. The purpose of this paper is to describe the development and implementation of a fully online research practicum as part of the medical school curriculum.

Methods: A fully online research curriculum for preclinical medical students was developed and implemented by the researchers as a mandatory component of student training. This curriculum addressed research education challenges in medical schools with limited resources and/or for situations for which face-to-face research may be impractical or expensive. Students were also encouraged to practice critical thinking and critical appraisal of the evidence. Student-initiated research projects were conducted in teams and completed with support from faculty research mentors and librarians. During the first two cycles, 86 student team projects were completed. During the third cycle, 45 team projects were conducted.

Results: Two complete cycles of a two-semester research practicum were completed. The majority of these studies were presented at regional, national, and international conferences, published as posters, and/or published as articles in peer-reviewed journals. Faculty mentor publications also increased as a result of engagement in the practicum.

Discussion: The results of this research practicum provide evidence of the usefulness of a hands-on tailored research program delivered online for increasing research literacy, promoting interest among medical students in research, and motivation for future engagement in research as verified by student project outcomes during and beyond the practicum experience. The practicum can be used for individual students or as a team-based approach. A research practicum such as this also has the potential to improve the prospects of a successful application for post-graduate training, grants, and high-impact publications.

## Introduction

Physician-scientists are MDs or DOs who undergo additional training in research and dedicate a significant portion of their time to pursue new knowledge about the delivery of patient care, disease, and health through research. Research is a critically important skill for medical trainees that helps physicians to advance the state of science and provide optimal healthcare to patients. Expediting physician-scientist training has thus become critical given the stagnation of clinical investigators in practice [1], regardless of the growing total number of physicians in practice, with the projected demand exceeding the expected supply [2-4].

Medical education strives to prepare students to practice medicine and provides the tools to do so successfully. Competing priorities, limited research faculty, shrinking research budgets, and meeting accreditation standards limit the amount of time and effort needed to train undergraduate medical students sufficiently for them to engage in independent research activities during graduate training and beyond. At most medical schools, a research “gap” year can be done after either the second or third year but taking the year off may not be logistically or economically feasible. Undergraduate research fellowships may also be available (the year between preclinical and clinical training) but are awarded to only a few students per class per year. These gaps delay graduation and separate the student from their known cohort support system.

Medical students’ exposure to research

Undergraduate medical students are incorporated into research in a variety of ways with no apparent consistency. Students may participate in summer research electives (one-two months), mandatory coursework, and extracurricular research activities. Students may be reluctant to take time out for research, particularly if they are not receiving school credit.

Nonetheless, even students not interested in a research career will obtain skills transferable to clinical practice [5]. Something as cursory as writing a literature review can help students develop information literacy, library searching skills, critical thinking skills, and critical appraisal of evidence skills [6].

Another path for engagement in research is faculty-initiated studies. Students often "assist" the faculty in their ongoing research such as helping with lab work, writing up case reports, or conducting literature reviews for future papers. For several reasons, students may not be added as authors on publications related to projects they have worked on (e.g., they were not given substantive tasks that warranted authorship, the project was put on hold or abandoned by the faculty, the student moved into their clinical rotations, authorship was not negotiated up front). However, some medical schools do not have ample faculty with (1) expertise in research and/or (2) time to devote to student research mentoring.

Factors affecting medical student research

There are published reports that assessed the competencies, interests, barriers, and facilitators of research participation among undergraduate medical students [7-20]. Common barriers include lack of knowledge, lack of time (competing priorities), insufficient funding, and lack of mentoring (e.g., limited access to research mentors in their areas of interest). Students may lack the confidence and/or self-efficacy, and thus the inclination or motivation to conduct independent research while in medical school [7,21-25]. These factors combined may define "research literacy" as a unique entity affecting a person’s ability to conduct research. Research literacy in this aspect goes beyond memorization and recall of facts.

Another factor that impedes medical students from being involved in research includes not having good role models or mentors [7,26]. Moreover, matching the right mentor with a student is central to ensuring that students are not just providing assistantship in lab work or data collection, but rather providing an intellectual contribution to research projects [7,26].

The college’s influence, including the type and length of research opportunities offered, may also be relevant factors regarding students’ choice to engage in research. Additionally, ethical approval processes (whether in terms of time or paperwork) are often a chief difficulty facing faculty advisors and students alike. Moreover, the relatively short duration of the undergraduate research experience places limits on its publication or citation potential.

The rationale for the development of the research practicum

The number of students who report interest in research may be higher than that of students who are actually involved in research projects [14]. Reasons for this phenomenon may be due, in part, to the shortage of opportunities to meet students’ interests, lack of funding, good mentorship, or inflexible curricula that leave little or no time for research [14,25,26]. The acknowledgment that barriers exist combined with students’ desire to learn research led to the creation of a curriculum-based, comprehensive research training for preclinical undergraduate medical students that is delivered fully online.

There is a demand for a unique balance between scientific inquiry and outstanding clinical training. With this in mind, the research practicum (herein referred to as the “Practicum”) contains activities to transform the manner in which students learn and prepare for careers in academic medicine or clinical practice in which scientific investigation is involved. These innovative efforts in any medical education curriculum can provide students with a more unique and flexible educational experience and accelerate the preparation of physician-scientists to become leaders in biomedical research.

In this paper, the development and implementation of a Practicum for all second-year students enrolled in the Nova Southeastern University Dr. Kiran C. Patel College of Osteopathic Medicine (NSU-KPCOM) Research Practicum are described. These activities include a series of didactic lectures combined with a mentor-driven, structured yet flexible Practicum to promote students’ research literacy and promote critical thinking skills. The Practicum can be done exclusively online using internet-based platforms typically utilized in modern medical schools such as Canvas and Zoom. Further, the Practicum does not incur any costs from students, faculty, or medical school administrators (aside from faculty percent effort) and thus no extracurricular budget is required. No textbooks are required.

To our knowledge, the NSU-KPCOM Research Practicum is the only one of its kind to provide a mentor-driven, comprehensive, and tailored research program delivered fully online for increasing research literacy, promoting interest among medical students in research, yielding publishable products, and increasing motivation for future engagement in research. The Practicum can be used for individual students or as a team-based approach. A Practicum such as this also has the potential to improve prospects of a successful application for post-graduate training, grants, and high-impact publications.

Program overview

In August 2020, the inaugural Practicum was delivered fully online as part of mandatory curricula programming for second-year students enrolled in the NSU-KPCOM. The Practicum provided mentor-driven, structured, interactive tailored training with student access to free statistical and data management software. All students participated in team research projects with an average of 8-10 students per team. Approximately 800 students have successfully completed the Practicum between 2020 and 2022.

The practicum protocol

A bottom-up approach to student participation in research (e.g., student-run, faculty-facilitated student research teams) is embraced by providing opportunities for research projects with a continued focus on sustained mentorship that includes advancing communication and clinical skills as well as professionalism - in tandem with their academic studies. All second-year students are required, as part of the medical school program, to enroll in the Practicum that (1) involves students in the practicalities of research, from planning to execution, by providing opportunities for them to participate in team-based research projects; (2) incorporates the teaching of key research methodologies learned in didactic courses provided in the curriculum for students to apply to tangible team-based research projects; (3) facilitates the generation of new research questions by students; (4) provides research mentors from the college and the wider research community at the university or affiliated hospitals to help facilitate their research activities; and (5) supports students to present and publish their research.

The Practicum addresses key elements in designing and conducting health and epidemiological research, including (1) understanding research theory and practice; (2) evaluating research through reading, reviewing, summarizing, and assessment; (3) formulating good research questions; (4) conducting literature reviews; (5) selecting research approaches; (6) documenting, presenting, and writing the research topic and results; (7) exploring ethical issues in research design (plagiarism, human subject ethics, interpreting results); and (8) conducting a team research project.

The Practicum helps develop students’ abilities by increasing their research literacy, i.e., the capacity to obtain, process, and understand basic information needed to make informed decisions about conducting research. Research literacy in this aspect goes beyond memorization and recall of facts. By involving students in the logistics of research, from planning to implementation, their scientific acumen as well as their self-efficacy increases. In this way, by promoting research readiness in students transitioning to their clinical phase of training, graduates can begin residency, a generally stressful time, feeling confident if not masterful in their ability to conduct research.

The student-initiated research projects were conducted in teams with guidance and support from faculty mentors. The faculty mentors were particularly instrumental in promoting the academic medicine mission of training the next generation of physicians to engage in research to inform advancement and innovation in healthcare delivery. Due to specific circumstances, however (e.g., large class size, pandemics), the course directors needed to adapt the Practicum in the following ways.

Large Class Sizes

While small student research teams may be optimal (2-5 students), the medical college has approximately 400 students in each cohort and encompasses two campuses in Florida nearly 300 miles apart (i.e., Fort Lauderdale and Clearwater). Due to the large student body, students carried out team science with a faculty research mentor, with each group consisting of 3-10 students.

COVID-19 Pandemic

While the onset of the COVID-19 pandemic offered new opportunities for research, the logistical challenges in complying with coronavirus safety restrictions called for creative modifications of proposed research projects. What might have been a study conducted with patients face to face became one that utilized Internet-based technology such as Zoom for interviews, REDCap data management software for online surveys, and Rayyan systematic review collaboration software.

Flexible Design Based on Student Preferences

Based on student feedback, the curriculum was revised to teach only systemized review studies (e.g., systematic, meta-analysis, and scoping reviews) and is the focus of this protocol. These adaptations yielded excellent results while simultaneously highlighting the importance of critical thinking, flexibility, and creativity in scientific endeavors. This gave students more time to work on projects without having to wait for IRB approval and to finalize data collection, which could take several months. Moreover, data collection efforts sometimes yielded insufficient sample sizes leading to non-publishable papers. Lastly, there was a scarcity of faculty members with statistical analysis training to help analyze the results. The resulting change left more time to focus on publications of students’ work before the Practicum ended.

## Materials and methods

Internal support

Getting Started with Faculty

After faculty research mentors were identified, they attended an online synchronous orientation session that explained the goals and objectives of the Practicum, expectations of mentors, course content, student assignments, grading procedures, and a tour of the online learning platform (e.g., Canvas). Mentors were expected to attend all synchronous student sessions, but all sessions were recorded for future viewing for mentors who were unable to attend.

External support

Librarians

Finding good, reliable research sources is often challenging in academic health professions settings. Librarians provided instrumental help to students, particularly in developing a search strategy for systemized reviews, working with reference management software packages (e.g., EndNote), and citing good sources for their introductory literature reviews.

Statistical Consulting

For the meta-analysis, faculty members with statistical expertise were utilized as secondary mentors for projects in which the primary mentor did not have quantitative statistical skills.

Administrative

The medical college administration offers the use of (at no charge to students or faculty) Rayyan (https://www.rayyan.ai/), an online software platform to organize, manage, and accelerate collaborative systematic and scoping reviews, and EndNote, a reference manager.

The curriculum

The Practicum outlines the fundamentals of conducting research for medical students at the preclinical level. The sessions include the following general topic areas related to conducting systemized review studies (e.g., scoping reviews, systematic reviews, meta-analyses): choosing a research topic, developing a good research question, writing a literature review, developing a research protocol, collecting data, conducting the analysis, reporting the results, writing the discussion, scientific writing skills (abstracts, posters, manuscripts), and using research tools (Rayyan, EndNote referencing manager, online library databases).

Module “Start Here” for students

Getting Started with Students

In Canvas, a module labeled “Start Here” was created for students. Here is where students found dates and links to synchronous Zoom sessions (six total per Practicum), research faculty mentor bios with their research experience and interests, assignments and grading system, grading rubrics, the syllabus, information on plagiarism and academic misconduct, faculty virtual office hours, and other logistical information. Students were instructed to self-assign to pre-constructed groups in Canvas based on their research interests and mentor preference; each group was labeled by mentor’s name and had a limit on the number of students who could self-assign predetermined by the course directors in coordination with the mentors.

Module "Start Here" for mentors

Getting Started with Mentors

A mandatory orientation session with mentors before the start of the Practicum was accompanied by a Canvas module designed to offer training and resources for mentors and was appropriately labeled “Mentor Center.” The module was visible only to the faculty research mentors. Sample topics included: helping students define their review question and inclusion criteria, Rayyan video tutorial, navigating the Practicum using an online learning platform (i.e., Canvas, Blackboard), course requirements, how to grade group assignments, and grading rubrics. Other logistical information (e.g., assignments and due dates, student emails by group) were also provided as well as PowerPoint presentations and recordings from synchronous Practicum sessions with all students. The Mentor Center was updated periodically as needed.

Sample learning modules for meta-analysis, systematic, and scoping reviews

This curriculum was conducted over two semesters (15 weeks each semester). Each module covered 2-3 weeks of material, with each week covering a different topic that builds on itself. At the end of each module is an exercise (graded as a group assignment) focused on completing the steps for conducting the review study. To ensure engagement on the individual level, there was an article screen assignment in Rayyan and a series of quizzes throughout the semester that tested students’ critical thinking skills related to research methods and appraisal of the scientific literature. Students were also given access to plagiarism detection software to use before submitting assignments if applicable. Grading rubrics were developed and made accessible so both students and mentors understood the expectations for all deliverables. Table 1 delineates the outline of modules pertaining to conducting systematic/scoping reviews and meta-analysis studies.

**Table 1:** Modules in the Research Practicum

Module	Main Topic
1	Getting Started With Your Research Project
2	Preparing and Writing the Background/Literature Review
3	Developing Your Research Protocol
4	Data Collection (Screening and Tracking Articles with Rayyan)
5	Data Extraction/Writing up the Methods Section
6	Critical Appraisal of Articles and Finalizing the Data Selection
7	Analysis/Presenting the Results (With PRISMA Diagram and Final Summary Table)
8	Writing the Discussion and Conclusion
9	Dissemination Plan
10	Writing Abstracts
11	Creating Posters and Preparing Oral Presentations
12	Finalizing the Manuscript

Each module contained the following components

Module Objectives

A concise list of the learning objectives of the module that are reflected in the information and activities throughout the module.

Asynchronous Virtual Seminar

This page included the module’s learning objectives and information in written form on the module's topic. It included a module overview and week-by-week detailed explanations, relevant attachments, definitions, examples, and other information pertinent to the module topic. This was an economic and effective instructional tool for teaching students who are at a distance from their instructor.

Supplemental Learning Materials

Included in each module were video tutorials (from reputable lectures found online or pre-recorded lessons developed by the course director, links to relevant websites), annotated PowerPoint presentations, and relevant scientific articles.

Links to Assignments

Links to the module’s quiz and assignment (including examples of completed assignments) were included in each module.

Communication

An online discussion forum labeled “Ask Your Professor” was available for students to post questions pertaining to the corresponding module. Students were also provided the course directors’ emails and an online scheduling assistant, Calendly, with a Zoom meeting link to set up appointments with the directors (individually or as a group).

Evaluations

At the midpoint and conclusion of the Practicum, students completed a survey evaluating their mentor. Feedback provided by students was instrumental in evaluating mentor performance and engagement. Mentors who were not available to meet with their research team(s) regularly or who did not provide adequate guidance were replaced (after attempts at remediation) and not asked to serve the following year. Course evaluations were conducted at the end of the Practicum (end of the second semester) via the institution in which students provided quantitative and qualitative feedback regarding the course directors and the course itself.

## Results

The Practicum cultivated engagement in research throughout the second year of medical school by integrating a curriculum that provides not only education in research methodology, but tangible and tailored opportunities for student research. It was cost-effective, with the allocation of faculty effort as the only financial expense.

Student team projects from years 2020-2022 consisted of collecting data through questionnaires and interviews, conducting quasi-experiments, preparing systematic and scoping reviews, retrospective medical chart reviews, and analyzing large healthcare data sets. These adaptations yielded excellent results while simultaneously highlighting the importance of critical thinking, flexibility, and creativity in scientific endeavors.

Table 2 reports the number of Practicum student projects by year completed.

**Table 2:** Student Accomplishments (Working in Teams of 2-10 Students)

Year	Number of Students	Number of Projects Completed
2021	402	52
2022	396	34
2023	392	45 (projected completion 2023)

The student projects were disseminated as published abstracts, published posters, articles in peer-reviewed scientific journals, international conference proceedings, and presentations at regional and national scientific conferences. Many of the posters were presented at local or school-initiated student poster competitions, with several taking away awards. For each publication, faculty mentors were listed as senior (last) authors, increasing faculty research output in addition to teaching load or service (i.e., student mentoring or advising) to the college.

Table 3 reports the titles of the Practicum student team projects completed in 2021.

**Table 3:** Practicum Student Team Projects Completed in 2021 (N=52)

Topic Title
A Literature Review of Selected Sexually Transmitted Infections in the Time of COVID-19
A Review of Petroclival Meningioma’s and Trigeminal Neuralgia
A Scoping Review of COVID-19 and Stroke Risk Among Young Adults
A Scoping Review of the Role of Telemedicine in Pediatric Type I Diabetes Mellitus
A Study on the Effects of COVID-19 on Dietary Intake and Exercise in College Students
An Approach Toward Assessing Head-and-Neck Lymphedema Using Tissue Dielectric Constant Ratios: Method and Normal Reference Values
Assessing the Effectiveness of a Brief Synchronous Online Training Program for Health Professions Students on Providing Optimal Clinical Services for Victims of Human Trafficking
Assessing the Use of Telemedicine Among Patients Seeking Health Services During COVID-19
Assessment of Quality-of-life Reporting After ACLR in Division 2 Athletes
Attitudes of Muslim-American Women Towards Oral Contraceptive Pills and the HPV Vaccine
Attitudes Towards End-of-Life Care Planning During COVID-19 in a Sample of the General Population in the U.S.
Barriers to Medical School Matriculation Amongst Black Males: A Scoping Review
Can the ORT, SOAPP, or PROMIS Predict Opioid Consumption Following Shoulder Surgery?
Comprehensive Look at Sacral Dysmorphism and a Review of Altered “Safe Zones” in Sacroiliac Screw Fixation: A Scoping Review
Confounding Factors That Can Be Attributed to the Rise and Fall of COVID-19 Incidences Within Florida Counties
Contradictory Effects of TNFi Treatment on Cardiovascular Health in Patients with Rheumatoid Arthritis
COVID Cases and Demographic Data by ZCTA (Zip Code) in Broward and Miami-Dade Counties
COVID-19 Anxiety and Personal Protective Behaviors in the General U.S. Population
COVID-19 Misinformation in Social Media: A Scoping Review
Effects of COVID-19 on Medical Students' Study Habits, Academic Performance, Home Life, Quality of Sleep, and Spending Habits
Effects of COVID-19 on Mental Health, Hygiene Behavior, Nutrition, Weight Status, Physical Activity, and Exercise Status
Elevated Cytokine Levels as a Cardinal Feature of Respiratory Pathogenesis in COVID-19 Patients: A Scoping Review
Experiences Regarding COVID-19 Pandemic Among Medical Residents
Faculty Attitudes and Self-efficacy Towards Online Teaching
Florida Healthcare Providers' Assessment of Primary Care Visits During the COVID-19 Pandemic
Fourth-Year Medical Students’ Motivations, Perceptions, and Intentions to Train in Residency Programs with Medically Underserved Populations During the COVID-19 pandemic
Garden Based Nutritional Interventions: A Scoping Review
Healthcare Professional and Patient Attitudes Toward COVID-19 Safety Protocols
Lecture Modality and Student Academic Success
M2 Students Lecture Modality and Personality Type
Osteopathic Manipulative Techniques and Women During Pregnancy: A Scoping Review
Osteopathic Medical Students Attitudes and Psychosocial Factors as Predictors of Perceived EMR Usefulness and Ease of Use
Osteopathic Medical Students’ Perceived Satisfaction Regarding Anatomy Lab: Virtual vs Cadaver
Patient Experience and Attitudes Towards the Osteopathic Manipulative Treatment (OMT)
Perceived Stress and Caffeine Intake Among Students in U.S. Medical Schools
Predictors of Self-reported Life Stressors in Preclinical Osteopathic Medical Students During COVID-19
Racial Disparity in Maternal Mortality: A Scoping Review
Sleep Deprivation and Academic Performance and Learning in Medical Students and Residents: A Scoping Review
Stem Cell-Based Therapies for Treatment of Neuropathic Pain: A Scoping Review
Student Perspectives on Medical Education Quality Pre- and Post-COVID-19
Survey of Medical Student Behavior in transition to Online education During Viral Pandemic
Sympathetic Innervation of Lacrimal Glands: Textbook Contradictions and Long-Lasting Conundrum in Research Literature
Targeting Aging with Curcumin: A Scoping Review
Telemedicine and Targeting Medication and Treatment Adherence in Diabetes Mellitus Type 2 Patients: A Scoping Review
The Association of Personal Characteristics and Experiences to COVID-19 Protective Attitudes and Behaviors
The Association of Stress, Collaborative Learning, Social Presence, and Social Interaction with Teaching Modality Type
The Effects of COVID-19 on Physicians’ Perceived Ability to Provide Care for Patients with Type II Diabetes Mellitus
The Impact of Copper in Development and Progression of Alzheimer’s Disease: A Scoping Review
The Impact of Racial Discrimination and Disparities on the Medical Treatment of African American Women: A Scoping Review
The Potential Link Between Type Two Diabetes Mellitus and Alzheimer’s - A Scoping Review
United Network for Organ Sharing Database Analysis of Factors Associated with Kidney Transplant Time on Waiting List
Videogames and Robotic Surgical Competency: A Scoping Review

Table 4 reports the titles of the Practicum student team projects completed in 2022.

**Table 4:** Practicum Student Team Projects Completed in 2022 (N=34)

Topic Title
A Systematic Review of Therapeutic Options for Pancreatic Ductal Adenocarcinoma
A Scoping Review of Outcomes of Disseminated Intravascular Coagulation In Oncologic Pediatric Patients
The Therapeutic Effects of Ketamine in The Context of Posttraumatic Stress Disorder, Depression, and Substance Use Disorder: A Narrative Review
The Impacts of Telehealth and Telemedicine on Access to Primary Care Specialists in Underserved Populations During COVID-19: A Scoping Review
The Impact of COVID-19 on Kidney Transplant Patients in the United States: A Scoping Review
The Effect of Discrimination on Cortisol Levels: A Scoping Review
The Impact of the COVID-19 Pandemic and Patient Compliance with Breast, Colon, and Prostate Cancer Screening: A Scoping Review
The Use of Improvisational Music Therapy in Improving the Quality of Life of Children with Autism Spectrum Disorder: A Scoping Review
Efficacy of Vitamin C and Vitamin D in the Treatment of COVID-19: A Systematic Review
Bias and Lack of Education as Causes for Increased Mortality in Patients of Color with Melanoma: A Scoping Review
Telemedicine ICU Implementation During COVID-19: A Scoping Review
Therapeutic Impact of Exercise in Patients with Psychiatric Illnesses: A Scoping Review
A Scoping Review of the Effects of a Gluten-Free Diet on the Neurological Manifestations of Celiac Disease
Advantages of Diverse Skin Cancer Prevention Interventions: A Scoping Review
A Descriptive Cross-Sectional Study of Coping Behaviors of Students in The Healthcare Professions During COVID-19
Placental Pathology Due to Maternal Covid Infection and its Effect on Neonatal Outcomes: A Scoping Review
The Effect of the Mediterranean Diet on Rheumatoid Arthritis: Systematic Review
Treatment Outcomes for Type II Diabetes (T2D) Patients Hospitalized with COVID-19: A Scoping Review
Is There a Relationship Between the Gut Microbiome and Inflammatory Lung Conditions? A Scoping Review
The Relationship Between Obsessive-Compulsive Behaviors and the Gut Microbiome: A Scoping Review
A Scoping Review of the Effects of Dietary Strategies on Risk and Neuroprotection in Alzheimer’s Disease
Alterations in Gut Microbiome of Parkinson’s Disease Patients: A Scoping Review
The Relationship Between Gut Microbiota and Sleep in the General Population: A Scoping Review
Using Medical Marijuana to Treat Postpartum Depression: A Scoping Review
A Systematic Review of Nontraditional Treatments for Post-Traumatic Stress Disorder
The Role of Aspirin, Statins, Colchicine, and IL-1 inhibitors in Primary vs. Secondary Prevention of Cardiovascular Events
Treatment of Stroke in Tuberculous Meningitis (TBM): A Scoping Review
Evaluation of Antidiabetic Drugs in the Prevention of Alzheimer’s: A Scoping Review
Temporal and Depth Variations in Skin Water Assessed via Skin’s Tissue Dielectric Constant Intra-Day and Day-to-Day Variations of Biophysical Skin Properties
The Effects of Osteopathic Manipulation on the Immune System
Efficacies of Alternative Therapies for Patients With ADHD: A Systematic Review
The Novel World-Wide Adaptations of Diabetic Management in the Face of COVID-19 and Socioeconomic Disparities: A Scoping Review
Psychoactive Drugs in the Management of Post-Traumatic Stress Disorder: A Promising New Horizon

Table 5 reports the titles of the Practicum student team projects in progress (N=45) at the time of this publication. The expected date of completion for these projects is April 2023.

**Table 5:** Practicum Student Team Projects in Progress (N=45)

Topic Title
Transjugular Intrahepatic Portosystemic Shunt (TIPS) vs Endoscopic Therapy Plus Pharmacotherapy
Complications and Adverse Events Related to Hyaluronic Acid Facial Dermal Filler Injections
Vaccination Status Through Radiographic Findings in Patients Who are Unvaccinated Against SARS-CoV-2 Regarding Severe Forms of Pneumonia
Examining the Role of Foods with Anti-Inflammatory Properties on Microglia Pathogenesis in Alzheimer’s Disease
Use of Male Contraception in Preventing Unwanted Pregnancy
Impact of Maternal Medical Cannabis Use on a Fetus
The impact of the COVID-19 Pandemic on Syringe Service Programs in the United States
Diagnostic Criteria and Screening Tools to Identify the Presence of Malignant Psoas Syndrome in Urogenital Cancer Patients
The Effects of Prescribed Medical Cannabis on Adolescents in the U.S.
Utility of Medical Cannabis in Treating Symptoms of Newly Acquired Fibromyalgia in Adults
Pain Management Protocols and Intrauterine Devices (IUD)
Emerging Non-Medical Interventions for Preoperative Anxiety in Pediatric Populations Undergoing In-Patient Surgical Procedures in U.S. Hospitals
Interventions for Pregnant Women with Systemic Lupus Erythematosus for Prevention of Premature Birth and/or Adverse Birth Outcomes in Newborns
Alternative Treatments Used for Children with Attention Deficit Hyperactivity Disorder
Health Disparities in Women Regarding Access to Care, Diagnosis, and Treatment/Management of Endometriosis in the U.S.
The Impact of Support During Childbirth on Birth Outcomes, Maternal Complications, and Recovery
Exercise Modalities to Improve Quality of Life for People with Dementia and Dementia Related Alzheimer’s Disease
Medical Implications of Abortion Bans for Pregnancies with Fetal Anomalies
Determining Implications of Usage of Biologics for Ulcerative Colitis Treatment in Adult Patients with Diabetes
The Role of Mast Cells in Intracranial Aneurysm Pathogenesis
Manifestations of Marfan’s Syndrome
Patient Populations with Coronary Microvascular Disease and its Relation to COVID-19
Impacts of Skin Properties and Color on Noninvasive Assessment of Peripheral Blood Oxygen Saturation in Hypoxic Patients
Overview of Methods for Detecting Lower Extremity Edema, Lymphedema, and Lipedema
Bioelectric Property Measurements in Differentiating Benign versus Malignant Tumors in Humans
Evidence Based Impact of Static and Pulsed Magnetic Fields on Blood Flow and Vascular Complications
Benefits of Osteopathic Manipulation Treatment (OMT) in Post-surgical Patients
The Role of Hyperparathyroidism and its Related Peptides in the Development of Colorectal Cancer in Adults
DPP4-inhibitors and Alzheimer’s Disease
The Effects of N-acetylcysteine on Inflammation-related Outcomes in Polycystic Ovary Syndrome (PCOS)
Racial and Ethnic Health Disparities in Post-Partum Women with Diabetes in Rural, Suburban, and Urban Locations in the United States
Current Proposed Managements for Adult Patients with Bacterial Meningitis Simultaneously Affected by Stroke
Social Determinants of Health in Chronic Kidney Disease
Adverse Effects of Different Types of Anesthesia on Women’s Health Outcomes in the United States
Orthobiologics Used for Knee Pathologies in Adults
Chronic Extremity Edema Management
Relationship Between Enterotype and Metabolic Syndrome
Uses of Fecal Microbiota Transplants
Monkeypox Virulence and Clinical Presentation and Pathogenic Capability
Maternal Obesity Impact on Neonatal Diseases and Future Complications
Effects of Socioeconomic Status, Race, and Family Location on Access to Care and Outcomes in Children with Sickle Cell Disease
Long Term Health Complications of Post-Pubescent Women Anorexia Nervosa
Positive and Negative Effects of Creatine Supplementation
Treatment Options for Women with Polycystic Ovarian Syndrome (PCOS)
Algorithms Utilized by Mobile Applications Marketed as a Method of Contraception and/or Aid in Family Planning

## Discussion

The Practicum incorporated the teaching of key research methodologies into the curriculum and emphasizes the importance of evidence-based medicine (EBM) and the need to broach new questions. Expediting physician-scientist training such as this might help address the stagnation of physician-scientists in the face of the growing total number of clinically oriented physicians who practice [1-4]. The platform described in this paper involved students in the practicalities of research, from planning to execution. As with all new educational programs, strengths were acknowledged, and lessons were learned.

Strengths

More Opportunities for Collaboration

There were multiple advantages to a fully online research program. It provided many opportunities for students to participate in research within their undergraduate curriculum. Students from different regional campuses from the same school seamlessly collaborated on team projects. Research mentors from outside the college or university were enlisted.

Fiscal/Logistical Advantages

There was no need to schedule classroom time, and money was saved on overhead expenses. Additional expenses, such as transportation costs, were eliminated for both mentors and students. Also, no textbooks were required as all learning materials were provided online.

Flexible

The practicum was implemented during the second year of medical school but can be implemented for first-year students, depending on the particular school’s preclinical curriculum. Students had online access to the learning materials and mentors during off hours. The online Practicum was convenient, offered flexibility, and provided more individual attention.

Skills Acquired Through the Practicum Experience

Students developed research self-efficacy and confidence to conduct autonomous research [23]. The Practicum promoted time-management skills, making it easier to achieve optimal school-life balance. As a fully online program with minimal synchronous meetings with faculty, it helped students develop self-motivation and self-discipline while promoting teamwork and providing maximum flexibility for students to simultaneously complete their coursework.

Student Research Is Supported

The Practicum exposed students to the wider research community at the university. It encouraged them to appraise and respond to current research. Many published studies have reported on the importance of supporting student research in undergraduate medical school programs [7,15,17,23].

The Practicum aimed to support students in presenting and publishing their research, highlighting the potential career pathways within academic medicine. Students obtained skills such as writing a literature review and manuscript as well as library research skills [6].

Challenges

Creating and maintaining an online Practicum can present some challenges. Because the Practicum was offered in the second year, if a student failed, they would need to take the Practicum the following year. This would not allow the student to complete the preclinical requirements and thus could not advance to the clinical phase of their training (i.e., rotations).

To help ensure sustainability, the following issues need to be considered and several commitments from the administration need to be ensured. First, course directors must be flexible due to the competing interests of medical students (e.g., other course exams, natural disasters). Directors must also periodically check the mentors’ work for quality assurance of projects they are mentoring. Second, the school must be able to provide capable mentors in numbers that promote optimal ratios of mentors to students. Mentors must have time allotted and sanctioned by the administration, as much of the work occurs outside of synchronous class sessions. Mentor to student ratio may not be ideal in large classes or schools with few research faculty. Third, the school infrastructure, including technology provisions, must provide support in the way of free software for students (e.g., SPSS, REDCap), librarians, statistical faculty/staff, and an amendable IRB. Fourth, the school should provide sufficient venues (e.g., school-initiated conferences) for students to present their work. Ideally, the school should provide research funding to students for conference attendance and for students to present and/or publish their work in open-access journals, though this is not always feasible with the competing demands and strains on present-day college budgets. Last, offering students mentoring in different types of research (e.g., surveys, quasi-experiments, secondary data studies, and systemized reviews), as we had done with the first cohort, is time intensive. It requires creating additional learning materials and tailored didactic lessons for each type of study. Moreover, mentors with expertise in these research areas need to be accessible.

Implications for medical education

It is important to encourage participation in research throughout the preclinical years by integrating a Practicum that offers education and opportunities for research. When students understand the practicalities of research, they can feel more confident in their abilities [22,25] which could in turn promote research activity among medical students. Medical school faculty members should promote the advancement of the scholastic medicine mission of preparing future physicians to engage in research. This will help ensure advancement and innovation in healthcare delivery. Cultivating medical students’ engagement in research early on in their training should be a major goal for medical educators. There is a place for input across the course of the medical degree and that stepwise encouragement that is tailored to the interests, time resources, and career aspirations of the student that we believe will prove most beneficial.

## Conclusions

Undergraduate medical education strives to instill the competencies required for graduates to deliver safe, effective, and high-quality patient care. However, competing priorities, limited research faculty, and meeting accreditation standards have traditionally limited the amount of time and effort allocated to train medical students sufficiently for them to engage in research activities. As board exams have transitioned to pass/fail, student research is likely to become an increasingly important component of well-rounded medical student portfolios to increase their chance of successfully matching to competitive residency programs. One way to foster this process is to implement a fully online Practicum that is economical and easy to administrate such as the one described here.
